# Ethical and Practical Considerations in Implementing Population-Based Reproductive Genetic Carrier Screening

**DOI:** 10.3390/genes16040423

**Published:** 2025-03-31

**Authors:** Eva Van Steijvoort, Pascal Borry

**Affiliations:** Centre for Biomedical Ethics and Law, Department of Public Health and Primary Care, KU Leuven, Kapucijnenvoer 7 bus 7001, 3000 Leuven, Belgium; pascal.borry@kuleuven.be

**Keywords:** genetic carrier screening, reproduction, ethics, implementation, informed decision-making

## Abstract

Reproductive genetic carrier screening (RGCS) has emerged as a promising tool for identifying couples with an increased likelihood of conceiving a child with an autosomal recessive or X-linked genetic condition. By enabling early detection, RGCS has the potential to support informed reproductive decision-making. Historically, carrier screening initiatives aimed to decrease the prevalence of specific genetic disorders by targeting particular high-risk populations. More recently, there has been a shift towards offering RGCS for a wider range of conditions, with the goal of enhancing reproductive autonomy by facilitating informed decision-making and addressing inequities in access to healthcare interventions. However, this shift towards a more inclusive, population-based approach has raised questions about the tension between individual autonomy and public health goals, as well as concerns regarding the potential negative effects of large-scale genetic screening initiatives. Furthermore, there is growing interest in utilizing RGCS data for broader purposes, such as population-based genetic screening programs for hereditary cancers or identifying causes of unexplained infertility, which may present additional ethical considerations. This review explores the complexities surrounding the implementation of RGCS, with an emphasis on its objectives, the significance of informed decision-making, and the wider societal challenges it may present. By analyzing these interconnected factors, we aim to provide a thorough understanding of the potential implications of RGCS on both individual autonomy and societal dynamics.

## 1. Introduction

### 1.1. Reproductive Genetic Carrier Screening

Reproductive genetic carrier screening (RGCS) allows for ‘the detection of carriers of autosomal recessive and X-linked conditions in individuals or couples who do not have an a priori increased likelihood of being a carrier based on their or their partners’ personal or family history’ [1]. RGCS can be offered either before conception or during pregnancy as part of a population-based screening program or on an ad hoc basis. Based on the carrier status information provided, carrier couples may consider different reproductive options such as in vitro fertilization or intracytoplasmic sperm injection (IVF/ICSI) combined with preimplantation genetic testing for monogenic conditions (PGT-M), the use of donor gametes, adoption, or to refrain from having biological children together [1]. Carrier couples identified during pregnancy could also opt for prenatal diagnostic testing, such as chorionic villus sampling or amniocentesis, to confirm the presence or absence of the genetic condition in the fetus [2]. If the condition is identified in the fetus, couples may decide to either continue the pregnancy while preparing for a child with a monogenic condition or consider terminating the pregnancy [1]. Information gained through RGCS can also help to reduce the time required to diagnose a child with a genetic condition and could potentially inform the development of a targeted postnatal management plan, which may include treatment options or, when appropriate, palliative care [3].

Traditionally, RGCS has focused on more common conditions with a clearly defined phenotype, typically presenting in early childhood and associated with significant morbidity and/or a shortened life expectancy [1,4]. Early carrier screening initiatives targeted specific groups with an increased individual risk based on family history (e.g., cystic fibrosis (CF)), an increased population risk on the basis of ethnicity (e.g., Tay-Sachs screening in individuals of Ashkenazi Jewish descent), or an increased risk based on geographic location (e.g., β-thalassemia in several at-risk populations in the Mediterranean area) [3]. Previous research on these carrier screening initiatives targeting specific subpopulations has reported that individuals identified as carriers often experienced discrimination or social stigma due to their carrier status [1,5]. An example of this are the early mandatory screening programs for sickle cell anemia during the 1970s, where African-American carriers were denied health and life insurance or employment opportunities because their carrier status for sickle cell anemia was wrongly equated with having the condition itself [1]. However, several recent studies have shown that carrier screening based on ethnicity, geographic origin, or family history does not accurately reflect the true distribution of carrier frequencies for severe genetic diseases. Consequently, offering RGCS to all reproductive-aged individuals has the potential to improve the identification of more carriers/carrier couples, increase equity, and potentially reduce the risk of stigmatization of certain ethnic groups [5,6,7,8].

Simultaneous screening for a large number of conditions at a faster turnaround time became feasible following the introduction of new technological advances, like massive parallel sequencing (MGS), and a decrease in analysis and sequencing-related costs [3,9]. The development of the first commercial expanded RGCS product, which screened for 108 Mendelian conditions, was reported in 2010 [10]. Over the last couple of years, multiple providers have made expanded test panels available to the general population, which has resulted in a highly variable testing landscape [9,11]. Unlike traditional carrier screening, these expanded test panels screen all individuals for the same set of conditions regardless of family history, ethnicity, or geographic location. This approach eliminates the need for patients to have detailed knowledge of their family history or ethnic heritage [4].

A recent review by Delatycki et al. (2020) highlighted the significant variability in how RGCS is provided worldwide, largely influenced by geographical differences in carrier frequencies of genetic conditions, as well as local healthcare systems, financial considerations, and cultural and religious factors [12]. In most countries, RGCS is currently not available as an organized screening program but rather through opportunistic screening. For example, it is offered within the context of assisted reproductive healthcare. Furthermore, access to genetic counseling and the availability of reproductive options differ considerably [12,13].

A key debate is whether it is fair to offer RGCS selectively to some couples, particularly when it is proactively offered to those who are easily reachable or already engaged with healthcare services, while others may not be given the same opportunity. An additional concern is that high-risk populations, such as consanguineous couples or founder populations, may be overlooked in a population-based RGCS screening program, as specific genes or variants might not be included [1,14]. A more comprehensive risk assessment and investigation of familial variants is still highly recommended in this context to effectively address specific familial risks [4].

### 1.2. Guidelines and Recommendations for the Organization of RGCS

Professional organizations like the American College of Medical Genetics and Genomics (ACMG), the American College of Obstetricians and Gynecologists (ACOG), and the European Society of Human Genetics (ESHG) have published multiple recommendations about RGCS since 2013 [1,3,15,16]. These organizations have emphasized that RGCS should always be voluntary and based on an informed choice. Furthermore, there is a broad consensus that RGCS should ideally be offered before pregnancy, during the preconception period [1,4,17], and that the selection of condition-associated variants to be included in test panels should be guided by specific criteria [3,15,16,17,18].

There appears to be some consensus that RGCS panels should only include severe childhood-onset conditions with a well-defined phenotype, where the severity may impact reproductive decision-making. Many have advised against the inclusion of adult-onset conditions due to the possible violation of the minor’s autonomy and the right to self-determination [1,4,16,17,18]. However, in the absence of a clear legal definition for what constitutes a ‘severe’ genetic condition, its interpretation tends to be rather subjective [19,20]. There are also some ethical issues related to the classification of certain identified variants. Since not everyone with the same genetic variant will develop the same symptoms related to a particular genetic condition (reduced or incomplete penetrance) and since the same genetic condition can manifest differently among affected individuals (variable expressivity), it has been recommended to make the inclusion of certain conditions optional based on the principle of nonmaleficence [16]. Given the high complexity and uncertainty associated with some genetic variants, it is essential to balance the risk of conveying potentially burdensome or harmful information with the benefit of providing valuable insights for reproductive decision-making [13]. Therefore, it is important to emphasize that a false-positive result may mean additional uncertainty for the couple or could lead to unnecessary tests or interventions (e.g., prenatal diagnostic testing), while the choice to not report a potential condition-associated variant means that a couple might make reproductive decisions based on limited information [19,20].

In addition, it is well documented that large-scale human genomic studies have been predominantly performed on populations of European ancestry [21]. This bias has important implications because the under-representation of more ethnically diverse populations directly impacts our ability to translate genetic research into clinical practice. In the context of RGCS, this may result in inaccurate risk assessments of under-studied populations [22]. There is an urgent need to recognize the importance of studying under-represented populations to avoid health inequalities, as the benefits of genomic research are not distributed fairly. Moreover, this research could maximize the potential for new discoveries [21]. By including populations that reflect the full diversity of human populations in genomic studies, genomic variants associated with various health outcomes at the individual and population levels could be identified [23].

Professional guidelines have also emphasized that RGCS cannot replace risk-based screening for individuals with a family history of a genetic condition or for consanguineous couples [3,15]. Additionally, it cannot substitute for noninvasive prenatal testing (NIPT) or newborn screening. Instead, the role of RGCS should be viewed as complementary [1,15].

While RGCS is designed to provide individuals with genetic risk information, its implementation raises various ethical and societal concerns. This article explores the complexities of RGCS, focusing on its objectives, the significance of informed decision-making, and the wider societal challenges it presents. Through an examination of these interconnected elements, we seek to offer a thorough understanding of RGCS’s potential effects on both individual autonomy and societal dynamics.

## 2. The Aim of RGCS

Early carrier screening initiatives specifically aimed to reduce the prevalence of certain genetic conditions in communities with a high burden [1,24,25]. The focus on prevention within these initiatives stems from the public health paradigm, which typically focuses on improving the health of a population [24]. A well-known example is the reduction in incidence of Tay-Sachs disease (TSD) by more than 90% in the Jewish population in the United States and Canada following the introduction of a carrier screening program for TSD [26]. The rapid uptake and acceptance of this carrier screening program was influenced by several key factors, including the high carrier rate within Jewish populations, the development of community-based screening programs, the severity of the disease, advocacy from clinicians involved with at-risk families, and the support of Jewish organizations in coordinating early testing efforts [26]. Another long-established approach for detecting carrier status is cascade screening, which differs from population-based screening by specifically targeting individuals with a relevant family history (e.g., carrier testing for cystic fibrosis). In this setting, the emphasis is on individual and family outcomes, ensuring that those with an increased likelihood of conceiving a child with a genetic condition receive personalized information to guide their reproductive decisions. Due to its foundation in the clinical testing paradigm, this is not considered as a population-based intervention [24].

The transition to offer RGCS on a population-wide scale has sparked an ongoing debate about its fundamental objectives. The current view on the aim of population-based RGCS is predominantly informed by the clinical paradigm. This perspective assumes that the primary goal of RGCS is to enhance reproductive autonomy and support informed reproductive decision-making by identifying those couples with an increased likelihood of conceiving a child with an AR or X-linked condition [1,4,15,19,24,25]. Reproductive autonomy specifically refers to the capacity to reflect on one’s values and preferences (e.g., long-term goals) when making decisions about reproduction (e.g., when to become pregnant, whether to continue a pregnancy, etc.) [24]. Emphasizing reproductive autonomy may create the perception that RGCS is a clinical intervention. However, RGCS could also be seen as a public health intervention because of common features with other screening offers available to the public (e.g., testing of individuals without an a priori risk). While acknowledging that prevention of certain genetic conditions as a main goal for RGCS is problematic, because of the possibility of implicit judgments, it is important to acknowledge social and relational factors (e.g., socio-economic conditions) beyond an individual’s sphere of control that can undermine or limit reproductive choices [24,27]. Reproductive autonomy is rarely exercised in isolation in real-world contexts. Decisions about reproduction are often shaped by a variety of external factors, such as cultural norms, societal expectations, economic circumstances, and family influences. This understanding is crucial when addressing issues related to reproductive rights and decision-making, as external influences may conflict with a couple’s values and preferences.

Paradoxically, instead of strengthening reproductive autonomy, RGCS could potentially undermine it by creating pressures or limiting a couple’s choices [27]. The widespread implementation of RGCS could, for example, lead to the identification of a larger number of carriers and couples with an increased likelihood of conceiving a child with a genetic condition. This could drive greater demand for genetic counseling and more invasive confirmatory diagnostic tests. As these screenings and diagnostic tests become more normalized, it may place additional pressure on prospective parents to undergo RGCS, potentially creating expectations for participation in these screenings. On the other hand, it could be argued that there may be a reduction in the costs associated with the care and treatment of individuals living with certain genetic conditions if prospective parents choose preventive reproductive options. Given that, with the introduction of new and expensive therapies, the overall costs of treating patients with rare recessive diseases are expected to rise in the future [27,28]. However, this raises ethical concerns regarding reproductive autonomy, as financial considerations may influence reproductive decision-making, thereby challenging the extent to which individuals can exercise genuine freedom of choice.

Some have also criticized RGCS for being eugenic in its aim or possible outcomes. Eugenics refers to a range of practices that seek to improve the genetic composition of a population group/future generations by selecting desired heritable characteristics. The term immediately brings to mind a range of unethical programs (e.g., involuntary sterilization of individuals with genetic conditions) that were performed during the 20th century [29], the most notable example being the highly unethical activities undertaken under the Nazi regime in Germany. After the end of the Second World War, eugenics was widely condemned and remains strongly stigmatized, to the extent that is it often considered a taboo subject. In consequence, there is a significant reluctance to recognize the potential societal consequences of individual reproductive genetic decisions, including RGCS [29]. Although RGCS does not seek to alter or improve the genetic composition of the entire population, as some past eugenics programs aimed to do, it is important to acknowledge the potential effects of RGCS at the societal level. By improving informed reproductive decision-making, RGCS may lead to a decrease in the prevalence of certain conditions screened for, especially when carrier couples choose preventative reproductive options (e.g., pregnancy termination of an affected fetus, PGT-M, use of donor gametes, adoption, or not having biological children) [1,28]. In addition to emphasizing the voluntary nature of participation in RGCS and the freedom of choice, we must recognize the potential shift in societal norms that shape individual reproductive choices [29].

Although RGCS may not be specifically designed for individuals living with genetic disabilities or their families, it could still have significant implications for them. The disability rights movement has criticized RGCS because of its tendency to negatively shape public opinion about disabilities, the possible reduction in support for individuals living with certain genetic conditions, and a possible decline in public funding of treatments and cures for particular genetic conditions [30]. Nevertheless, others have also argued that offering RGCS more broadly could also reduce the risk of stigmatization and create more understanding and support for individuals living with certain genetic conditions [1,31]. As carrier screening becomes more widely known among the general population, awareness is likely to grow that all individuals are carriers of certain monogenic conditions [5]. Therefore, offering RGCS for monogenic conditions to all couples wishing to have children could potentially also help to normalize the understanding of carrier status and to reduce the stigmatization or discrimination of specific subgroups [8].

Even though it is not considered a primary objective, RGCS may also help reduce morbidity and mortality by enabling earlier diagnosis through close monitoring of children born to carrier couples. This, in turn, can facilitate the development of targeted management plans, including early therapeutic interventions [1,3]. RGCS can also be motivated by the desire to avoid painful experiences such as having to witness one’s child suffering or to grieve the loss of a child [19]. It has been argued that an approach with multiple compatible goals would be ethically acceptable, for example, increasing reproductive autonomy by facilitating informed reproductive decision-making and reducing inequities in access to healthcare interventions [24,32].

More recently, there seems to be a growing interest in utilizing RGCS genomic sequencing data for broader purposes to further boost knowledge and improve women’s health. Some have suggested using data from RGCS for population-based genetic screening programs for hereditary cancers or to enable genome-wide association studies focused on infertility phenotypes, such as predisposition to premature ovarian failure, increased risk of aneuploidies, complete oocyte immaturity, or blastocyst development failure [33,34,35]. While the preconception and pregnancy period could offer a unique window of opportunity to engage with women of reproductive age about genetic testing, questions arise as to what extent it is desirable to also offer these other screening/testing offerings in tandem with RGCS. While overlap does exist, there are also profound differences [33]. If healthcare systems and/or providers decide to offer this type of care model, it will require more careful consideration and extensive research to address concerns and uncertainties related to the complexity of providing genetic counseling, as well as the cost-effectiveness and feasibility of implementation. The implementation of these technologies must be approached with a high level of responsibility, ensuring that strong clinical evidence is established before they are integrated into routine practice [33,35].

## 3. Informed Choice and Informed Decision-Making

Some have questioned to what extent autonomous and/or informed choice is possible in a context where normative perceptions of people can be influenced when a certain practice becomes routine. As a result, people may regard RGCS as standard practice instead of an additional reproductive choice that is optional [31,36]. In addition, it has been suggested that offering RGCS for certain conditions is not a neutral activity, as it could be interpreted as a sign that some action should be taken when an increased reproductive risk is identified on the basis of the test results [19,37,38]. In this way, it could create pressure to prevent the birth of (possibly) affected children [17]. This may become even more contentious if RGCS is reimbursed by a health insurance provider. Since decisions can be influenced by many different internal and external factors, it is important to start from a place of a free, informed, and autonomous choice. This implies that every individual also has the right not to know and to decline an RGCS offer.

The autonomy of individuals or couples in making RGCS decisions should be preserved. Therefore, it is of utmost importance that those considering RGCS fully understand its purpose and potential implications. While genetic screening generally does not entail any physical risks, individuals should be aware that they may be faced with difficult choices, such as whether to pursue invasive diagnostic testing (e.g., amniocentesis) or whether or not to terminate a pregnancy.

As most autosomal recessive and X-linked conditions are rare, most individuals within the general population will not be aware of them. Therefore, more efforts are needed to increase awareness and to improve genetic literacy [39]. To address this, clear and transparent information should be made available to couples considering RGCS to reduce misconceptions and manage expectations. Providing information in advance could facilitate more efficient and effective pretest counseling, helping to avoid the potential risk of information overload [14]. While in the past, it was feasible to provide extensive information about a specific genetic condition, the expansion of test panels makes it practically impossible to discuss information about every genetic condition screened for. While less detailed information could possibly lead to a less informed choice, there is also the possible risk of ‘information overload’, which could undermine the aim of a meaningful choice in a reproductive context [1]. To mitigate this challenge, a generic consent process has been proposed [16,37]. This generic consent process focuses on broader concepts such as inheritance patterns, genetic screening issues (e.g., residual risk, variable expressivity, and reduced penetrance), reproductive options which could be considered, screening costs, and potential disclosure to other family members [37]. However, offering generic information should not be seen as a waiver of the patient’s right to more specific information [37]. Therefore, it is crucial to ensure that the option to request more specific and in-depth information remains accessible. Moreover, providing background information on how conditions and genes are selected, along with an updated list of screened conditions, should ideally be made publicly available. In the absence of relevant life experiences, attention should also be given to provide insights into the lived experiences of individuals and families impacted by genetic conditions [40]. This include perspectives on what it means to have the condition, to be a carrier, or to have a relative impacted by that condition [40].

Interactive multimedia tools, such as decision aids, have shown promise in enhancing understanding of theoretical concepts in a nondirective manner while promoting deliberation, particularly in resource-limited settings. Within the Australian Reproductive Genetic Carrier Screening Program, commonly referred to as “Mackenzie’s Mission”, the use of a decision aid facilitated discussions between couples and enabled more in-depth consideration of RGCS [41]. As such, interactive multimedia tools may serve as valuable tools in supporting informed decision-making and enhancing the feasibility of large-scale implementation of RGCS. To fully realize their potential, these tools must, however, be co-designed with end-users to ensure they fit into the care process and meet real-world needs. Furthermore, they should undergo proper evaluation to assess their effectiveness [42].

Finally, individuals identified as carriers as well as couples with an increased likelihood of conceiving a child affected by a specific monogenic condition should ideally receive comprehensive information and follow-up counseling, preferably provided by a trained genetic healthcare professional.

In the future, healthcare providers may also encounter increasingly complex decisions in the field of reproduction if more couples with an increased likelihood of conceiving a child with an autosomal recessive or X-linked genetic condition are identified prior to conception. While offering PGT-M for severe conditions such as cystic fibrosis is common practice, it may not be considered acceptable for some of the less severe genetic conditions that are included in RGCS test panels. Some healthcare professionals may be hesitant to pursue PGT-M for conditions they perceive as less severe or with manageable outcomes even though these conditions are identified through the expanded RGCS panels. It is possible that in some instances, this may not align with the preferences or values of prospective parents. Healthcare providers may find themselves navigating these differences, striving to respect the couple’s autonomy while also balancing their professional judgment about the risks, benefits, and ethical considerations of more invasive reproductive interventions.

## 4. Limitations

A limitation of this narrative review is that it lacks a systematic approach to study selection, which may result in the exclusion of relevant research. The process of selecting studies was guided by our judgment, potentially introducing bias. As a result, our conclusions are based on a qualitative interpretation of the literature. These factors should be considered when interpreting the findings.

## Abbreviations

The following abbreviations are used in this manuscript:

RGCS	Reproductive genetic carrier screening
TSD	Tay-Sachs disease
AR	Autosomal recessive
PGT-M	Pre-implantation genetic testing for monogenic conditions
